# Time series prediction of under-five mortality rates for Nigeria: comparative analysis of artificial neural networks, Holt-Winters exponential smoothing and autoregressive integrated moving average models

**DOI:** 10.1186/s12874-020-01159-9

**Published:** 2020-12-03

**Authors:** Daniel Adedayo Adeyinka, Nazeem Muhajarine

**Affiliations:** 1 Department of Community Health and Epidemiology, College of Medicine, University of Saskatchewan, Saskatoon, SK S7N 5E5 Canada; 2 Department of Public Health, Federal Ministry of Health, Abuja, Nigeria; 3 Saskatchewan Population Health and Evaluation Research Unit, Saskatoon, Saskatchewan Canada

**Keywords:** Sustainable Development Goals, Time series, Under-five mortality rate, Forecasting, Artificial intelligence, Deep learning, GMDH neural network, Autoregressive integrated moving average, Holt-Winters exponential smoothing, Nigeria

## Abstract

**Background:**

Accurate forecasting model for under-five mortality rate (U5MR) is essential for policy actions and planning. While studies have used traditional time series modeling techniques (e.g., autoregressive integrated moving average (ARIMA) and Holt-Winters smoothing exponential methods), their appropriateness to predict noisy and non-linear data (such as childhood mortality) has been debated. The objective of this study was to model long-term U5MR with group method of data handling (GMDH)-type artificial neural network (ANN), and compare the forecasts with the commonly used conventional statistical methods—ARIMA regression and Holt-Winters exponential smoothing models.

**Methods:**

The historical dataset of annual U5MR in Nigeria from 1964 to 2017 was obtained from the official website of World Bank. The optimal models for each forecasting methods were used for forecasting mortality rates to 2030 (ending of Sustainable Development Goal era). The predictive performances of the three methods were evaluated, based on root mean squared errors (RMSE), root mean absolute error (RMAE) and modified Nash-Sutcliffe efficiency (NSE) coefficient. Statistically significant differences in loss function between forecasts of GMDH-type ANN model compared to each of the ARIMA and Holt-Winters models were assessed with Diebold-Mariano (DM) test and Deming regression.

**Results:**

The modified NSE coefficient was slightly lower for Holt-Winters methods (96.7%), compared to GMDH-type ANN (99.8%) and ARIMA (99.6%). The RMSE of GMDH-type ANN (0.09) was lower than ARIMA (0.23) and Holt-Winters (2.87). Similarly, RMAE was lowest for GMDH-type ANN (0.25), compared with ARIMA (0.41) and Holt-Winters (1.20). From the DM test, the mean absolute error (MAE) was significantly lower for GMDH-type ANN, compared with ARIMA (difference = 0.11, *p*-value = 0.0003), and Holt-Winters model (difference = 0.62, p-value< 0.001). Based on the intercepts from Deming regression, the predictions from GMDH-type ANN were more accurate (β_0_ = 0.004 ± standard error: 0.06; 95% confidence interval: − 0.113 to 0.122).

**Conclusions:**

GMDH-type neural network performed better in predicting and forecasting of under-five mortality rates for Nigeria, compared to the ARIMA and Holt-Winters models. Therefore, GMDH-type ANN might be more suitable for data with non-linear or unknown distribution, such as childhood mortality. GMDH-type ANN increases forecasting accuracy of childhood mortalities in order to inform policy actions in Nigeria.

**Supplementary Information:**

The online version contains supplementary material available at 10.1186/s12874-020-01159-9.

## Background

Childhood mortality has traditionally been used as an important health indicator for assessing population well-being and consistently gained visibility in the Millennium Development Goals (MDGs) and Sustainable Development Goals (SDGs) [1]. It is a major public health threat in Nigeria and other low-middle-income countries (LMICs). Despite government efforts, the high under-five mortality rate (U5MR)—100 deaths per 1000 live births in 2017 [2], continues to burden the economic and health system of Nigeria.

In the absence of reliable vital registration system of under-five mortalities in most of LMICs, it is difficult for stakeholders to track progress towards achieving the child survival targets of SDG-3, which is aimed at reducing U5MR to 25 deaths per 1000 live births by 2030. To adequately plan for child survival programmes in Nigeria, large investment is required. In the face of the current economic situation of Nigeria, accurate forecasts of childhood mortalities will guide effective use of the limited health resources. On this note, sound modeling approach to improve childhood mortality estimates is needed in Nigeria. Considering the applicability of the traditional time series models for forecasting U5MR, there is little evidence to guide future planning of child health programmes in Nigeria. The argument is that, it is challenging for researchers to choose appropriate time series modeling techniques that can detect non-linear patterns of mortality rates [3–5]. However, some authors have proposed artificial intelligence such as deep learning techniques (e.g., artificial neural networks (ANN), convolution neural networks (CNN), recurrent neural networks (RNN)) [5–8] and machine learning techniques (e.g., support vector machine, random regression forest) [9–11] to improve accuracy of predictive models, while other studies have failed to demonstrate their suitability [12–14]. Unlike the conventional statistical/mathematical techniques such as Box-Jenkins approach of autoregressive integrated moving average (ARIMA) and Holt-Winters exponential smoothing method, ANN combines both linear and non-linear modeling properties [4, 5]. ANN closely follows the structure and functionality of the human brain and its neurons to solve complex problems faster with minimal human interventions, hence reducing error rates [6]. As ANN is evolving with newer algorithms, few studies [9–11, 15–18] have considered their applicability in population health studies. As far as we know, most of the studies in the fields of population health and medicine have used different deep learning techniques to optimize classification of health outcomes and medical data [19, 20], and disease screening/diagnosis [21, 22]. However, application of deep learning algorithm to forecast long-term childhood mortality is yet to be demonstrated in many LMICs (including Nigeria). Since childhood mortality data from resource-limited countries are often non-linear, noisy, and associated with large degree of uncertainties [2], forecasting with conventional statistical methods is somewhat difficult.

In the fields of engineering, agriculture, finance, and urban planning, group method of data handling (GMDH)—a type of artificial neural net—was observed to improve forecasting, compared with other neural networks. In a recent study, Ghazanfari et al [23] evaluated the performance of multilayer perceptron network (MLP)—a popular ANN algorithm, and GMDH-type ANN in predicting comprehensiveness and workability of concrete. Their study showed more accurate results with GMDH-type ANN. Also, other studies (outside of medicine and population health) have demonstrated the superiority of GMDH-type ANN compared with adaptive neuro fuzzy inference system (ANFIS) and long short-term memory (LSTM) [24, 25]. On this basis, our study focuses on generating accurate estimates and observing the patterns of U5MR for Nigeria during the SDG-implementation era. This study is in line with the 2014 United Nations’ call for data revolution of newer technologies that would improve data for sustainable development [26]. As new approaches are needed for child health programming in resource-limited countries like Nigeria, identifying and demonstrating the use of an appropriate model will ease application of long, time series data for monitoring the attainment of global framework indicators such as SDGs.

GMDH algorithm is a self-organizing inductive modeling and forecasting technique that extracts important information from the data to build a multilayered model through supervised learning [27]. A well-known problem with all time series methods, is that inadequately preprocessed input data can result in poor forecasting. Unlike the traditional statistical methods, no a priori knowledge of series stationarity and randomness is required for GMDH algorithm [28]. GMDH neural network can automatically learn from the data and uncover hidden processes not detectable by the conventional methods [29]. On the other hand, implementation of GMDH ANN turns out to be tricky because there is currently no theoretical guidelines for designing GMDH architectural layers in order to improve prediction accuracy [7]. Since, it is important to generate more accurate U5MR for Nigeria during the SDG-era, this study aimed to model long-term U5MR with GMDH-type ANN, and compare the forecasts with the most commonly used conventional statistical methods—ARIMA regression and Holt-Winters exponential smoothing models.

## Methods

This study was exempted from ethical review by the University of Saskatchewan Behavioural Ethics Committee (ID# 904) as it relied on a publicly available aggregated de-identified dataset [30]. The dataset used is the historical aggregated yearly U5MR of Nigeria for 1964–2017 (Supplementary file 1). The dataset was obtained from the official website of the World Bank [31]. The dataset was based on the reconciled country-level estimates from different data sources by the United Nations Inter-agency Group for Child Mortality Estimation team (UN IGME) [31].

We applied ARIMA regression, Holt-Winters exponential smoothing, and GMDH neural nets to predict annual U5MR. The historical mortality data span from 1964 to 2017, giving a total of 54 observations, which was adequate to fit ARIMA regression (i.e., at least 50 non-missing data points) [32]. Furthermore, we generated long-term forecasts to determine U5MR for Nigeria by 2030 (to coincide with attainment of SDGs). GMDH-type ANN was purposefully selected from the class of deep learning algorithms because of its robustness against incorrect, noisy, and small dataset [33]. Also, recent studies in other disciplines have demonstrated its superiority over RNN and LSTM [24, 25, 34, 35]. *P*-value < 0.05 and 95% confidence intervals (CI) were used to assess statistical significance.

### Fitting ARIMA model

**Fig. 1:**
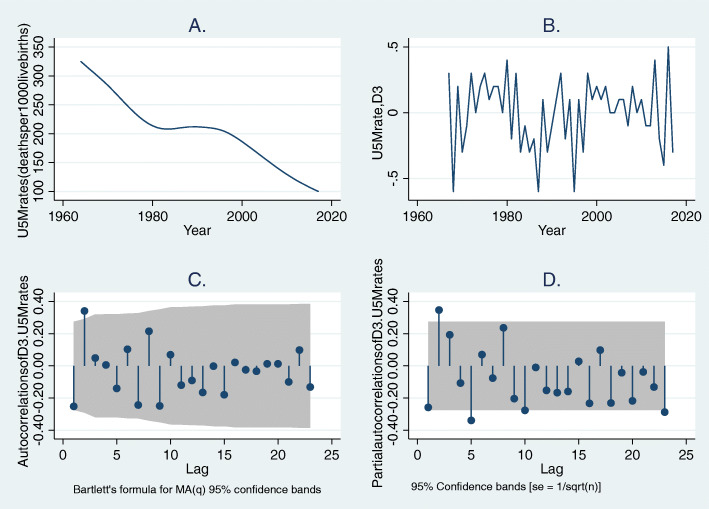
(**a**) Time series plot of under-five mortality rates in Nigeria for ARIMA modeling, 1964–2017 (B) Third order difference of yearly under-five mortality rates (**c**) Autocorrelation function plot of third order differenced under-five mortality rates (**d**) Partial autocorrelation function plot of third order differenced under-five mortality rates. *D3: third order differencing, U5M: under-five mortality, grey color in plots C and D: 95% Confidence Interval*

We utilized Stata™ version 15.1 software [36] to fit ARIMA regression model. The model construction is in four iterative steps: model identification, parameter estimation, diagnostic checking, and prediction. As the first step, data preprocessing geared towards understanding the underlying patterns in the data and data transformation was ensured. The stationarity of the aggregated U5MR was assessed by plotting line graph (Fig. 1a). It was observed that the assumption of stationarity for time series analysis was violated as evident by the non-seasonal downward trend of the overall under-five mortality rates. After different calibrations, third-order differencing was appropriate in removing the observed trend (Fig. 1b). Next, Dickey-Fuller (DF) test with drift was used to assess the stationarity of the differenced data (DF = − 9.02, lag order = 2, *p*-value< 0.001), and the absolute value of t-statistic was greater than the critical value at 5% level (9.02 vs. 1.68, p-value< 0.001).

The autocorrelation function (ACF) and partial autocorrelation (PACF) plots were also checked to determine the structure of the correlation between time lags of the differenced data (Fig. 1c and d). The ACF plot had a significant spike at the second lag, indicating second order moving averages (MA (2)) or ARIMA (0,3,2). Furthermore, ARIMA (0,3,2) model had the lowest Akaike’s Information Criteria (AIC) and Bayesian Information Criteria (BIC), hence it was considered suitable for fitting the actual under-five mortality rates. The model with smallest possible number of parameters (principle of parsimony) was selected to represent the distribution of the data. Also, the model comparison with AIC and BIC is valid, because the candidate models fitted the same data (U5MR) [37]. Following this principle, the specified ARIMA (0,3,2) model is expressed as:

1 $$ {\Delta }_3{y}_{t=}{b}_1\ast {\varepsilon}_{t-1}+{b}_2\ast {\varepsilon}_{t-2} $$

2 $$ {\Delta }_3{y}_t={y}_t-{y}_{t-3} $$

$$ {y}_t\  and\ {\varepsilon}_t\  were\ the\ actual\ value\ and\ random\ error\  at\  time\ period\ t, respectively.{b}_1\  and\ {b}_2\  where\ the\ model\ parameters\ of\ moving\ averages\  at\  lag\ 1\  and\  lag\  2\ \left(- 0.35\  and\  0.62\  respectively\right)\  with\ standard\ deviation\ \delta = 0.22. $$

**Fig. 2:**
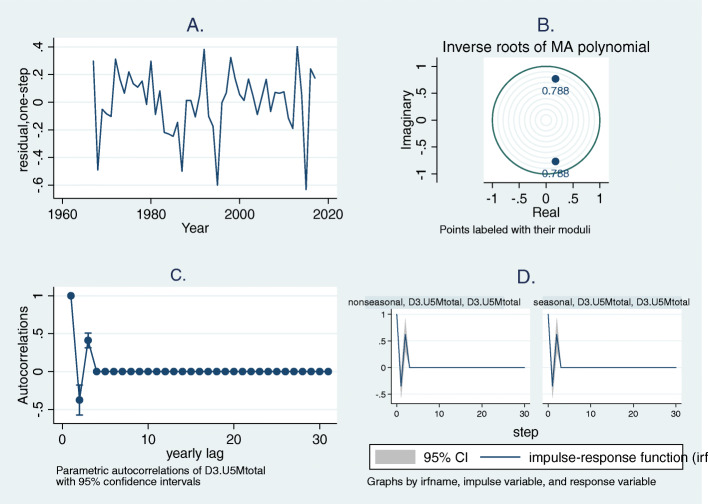
Diagnostic plots for ARIMA (0,3,2) of under-five mortality rates, Nigeria (1964–2017) (**a**) Residual plot (**b**) Inverse roots of MA polynomial (**c**) Autocorrelations of differenced rates (**d**) Impulse-response function plot

The adequacy of the fitted model was determined by the randomness of the model residuals (Fig. 2). Also, all the eigenvalues for stability of estimates were less than one and the inverse roots of MA polynomial visually indicates that the eigenvalues were within the unit circle (modulus of 0.79). This suggests that the MA parameters satisfied invertibility condition (Fig. 2b). This was further confirmed with Portmanteau (Q) test for white noise (Q = 32.3, *p*-value = 0.095).

### Fitting Holt-Winters exponential smoothing model

**Fig. 3:**
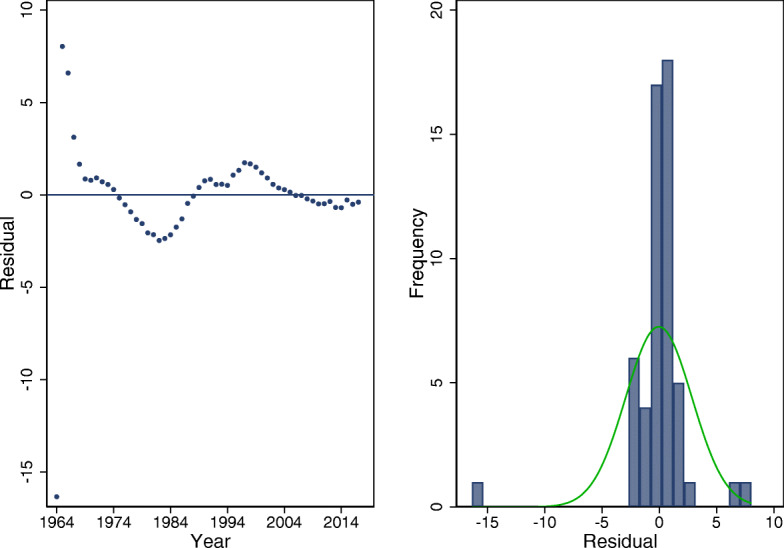
Residual plots for Holt-Winters exponential smoothing for overall under-five mortality rates, Nigeria (1964–2017)

Holt-Winters non-seasonal smoothing (often referred to as triple exponential smoothing) was used to predict the overall under-five mortality rates. According to Chatfield [38], it is the most advanced method in the category of smoothing methods. The smoothing parameters were automatically generated with Stata™ version 15.1 software [36] prior modeling with Holt-Winters method. The α (level) and β (slope) of trend should lie between 0 and 1, with values closer to 0 implying that the estimates at the current/future time points are based on recent observations [38]. The optimal smoothing weights were computed as α=0.91 and β=0.51. The residual plots after fitting under-five mortality rates for Nigeria, using Holt-Winters exponential model are shown in Fig. 3.

### Fitting GMDH-type artificial neural network model

The time series artificial neural network was implemented by the GMDH-type neural network core algorithm in GMDH Shell DS version 3.8.9 software [39]. We used the built-in time series pre-processing features of GMDH-type algorithm [40] to automatically remove the under-five mortality trend. The target variable (U5MR) was automatically transformed into cube root, with a minimum of zero lag and maximum of 6 lags. The input variables included time and lags of the transformed mortality rates. The polynomial neuron function of GMDH-type model is as follows:

3 $$ f\left({x}_i,{x}_j\right)={a}_o+{a}_{1.}{x}_i+{a}_{2.}{x}_j+{a}_{3.}{x}_i.{x}_j $$

$$ where\ x=\left({x}_i,{x}_2\dots \right), the\ input\ variables\ vector, and\ A=\left({a}_0,{a}_1,{a}_2,..\right)\  the\ vector\ of\ weights. $$

**Fig. 4:**
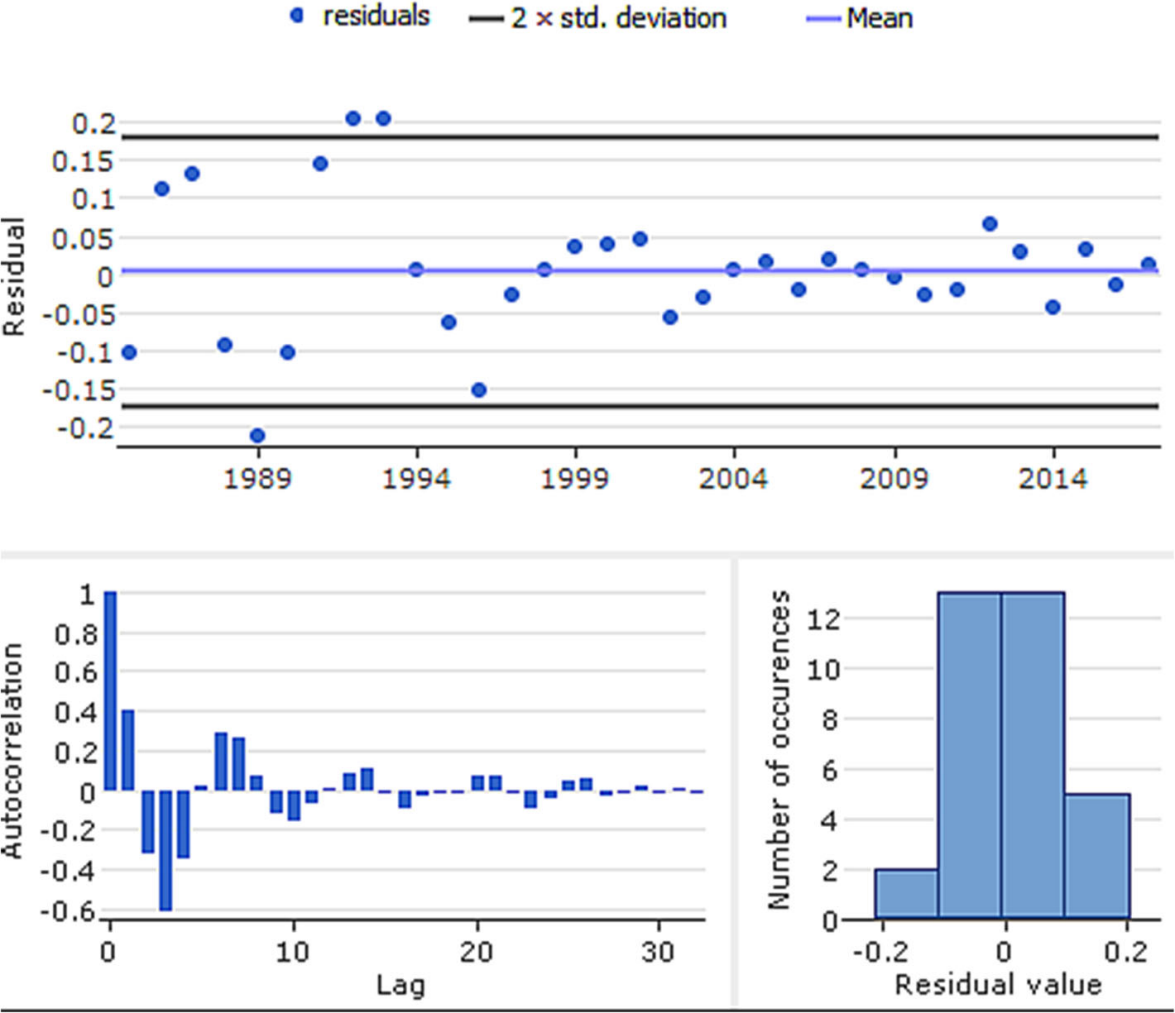
Residual plots for GMDH-type neural network for overall under-five mortality rates, Nigeria (1964–2017)

To avoid under/over-fitting of U5MR arising from improper training of dataset, the network was implemented with the dataset randomly partitioned using Pareto principle [41, 42]—80% was used for training and 20% was the test dataset for evaluating model accuracy. We designed an optimal neural-type time series model based on best performing hyper parametrization with polynomial neural networks of GMDH-type [43] (Fig. 4). Following the rule of thumb that the number of hidden neurons should be less than twice the input layer size, we developed the neural architecture [44, 45]. After different calibrations of neural architecture, the parameters for the network was configured with maximum number of network layers of 60 and initial neurons of 25. Similar to a method used by Banica et al. [46], we stopped creating new neural layers when:  (1) the new layer failed to improve the model accuracy, compared with preceding layer, (2) changes in testing error was less than 1%, and (3) configuration limit for the number of layers has been reached. Adequacy of the model was further confirmed with criterion value and residual plots (Fig. 4). With low criterion value of 1.01е-5, the final neural architecture adequately fits the data. The parameters and coefficients of equation for GMDH-type model are given as:

4 $$ {Y}_{\left[t\right]}=5.206{e}^{-05}-{N}_{25}\ast 0.077+{N}_2\ast 1.07734 $$

$$ Where\ Y\  corresponds\ to\ year\ of\ forecast, and\ N\  indicates\ neurons\  2\  and\  2 5. $$

### Model comparison

The foremost problem with measuring prediction accuracy is the identification of key performance indicators. Although mean absolute percentage error (MAPE), mean squared error (MSE) and RMSE are the commonly used accuracy metrics, they are prone to asymmetrical distribution of errors [47]. MAPE is generally not considered as a good performance indicator because of its disadvantages— (1) only accurate for ratio-scaled data, and (2) it disfavors models when the predicted values are more than the actual (historical) values [48]. On the other hand, a benefit of using RMSE is that it is more appropriate if large errors are anticipated. Even though strength of performance measurements vary, we selected root mean absolute error (RMAE) because of its robustness against outliers [49]. In addition, RMSE was chosen because it minimizes the effects of bias, and measures dispersion of prediction errors (i.e., model stability) [49]. The model with the lowest RMAE and RMSE values provides good fit for U5MR in Nigeria.

Furthermore, modified Nash-Sutcliffe model efficiency coefficient (NSE) was calculated to address the challenges of overestimating extreme values, arising from squared differences of actual and predicted values in original Nash-Sutcliffe efficiency equation [50]. Efficiency coefficient of ≥0.9 implies highly accurate prediction, and < 0.8 implies inaccurate prediction [51].^.^

Relative to the observed rates, statistically significant differences in loss function between forecasts of each of ARIMA and Holt-Winters models were compared to GMDH-type ANN, based on absolute value error from Diebold-Mariano (DM) test [52]. DM test is a statistical test for comparing two competing forecasts based on loss-differentials, given the historical (observed) values. In estimating the predictive accuracy, squared error was not used because of its tendency to overestimate errors [53]. Also for the long-run variance of the differenced series from its autocovariance function, a maximum lag order of 9 was selected by Schwert criterion and the weights of Bartlett kernel (i.e., zero autoregression).

The measurements are expressed as [54]:

5 $$ RMAE=\sqrt{\frac{1}{N}\sum \limits_{i=1}^N\mid {y}_i-\hat{y\mid }} $$

6 $$ RMSE=\sqrt{\frac{1}{N}\sum \limits_{i=1}^N{\left({y}_i-\hat{y}\right)}^2} $$

7 $$ modified\  NSE=1-\frac{\Sigma {\left|{y}_i-\hat{y}\right|}^j}{\Sigma {\left|{y}_i-\overline{y}\right|}^j} $$

$$ Where\ \hat{y}= predicted\ value\ of\ y,\overline{y}= mean\ value\ of\ y,j=1 $$

To further test the equivalence of the in-sample predictions (from the individual methods) with the observed historical values, Deming regression—an extension of errors-in-variables regression was performed. Deming regression assumes that forecasting errors are caused by the methods used [55, 56], (sample size > 20) [56], and the measurement error variance ratios (λ) are constant [55, 57]. When λ = 1, Deming regression is like orthogonal regression. An intercept (β_0_ or constant) with a confidence interval including 0 (i.e., intercept is not significantly different from zero) suggests no systematic difference/ bias between the measurements [56]. Also, slope coefficient (β_1_) with a confidence interval including 1 (i.e., slope is not significantly different from 1) indicates absence of proportional differences [56]. The Deming regression model utilized jack-knife replications to estimate the standard errors (SE) and 95%CI of the coefficients.

## Results

**Fig. 5:**
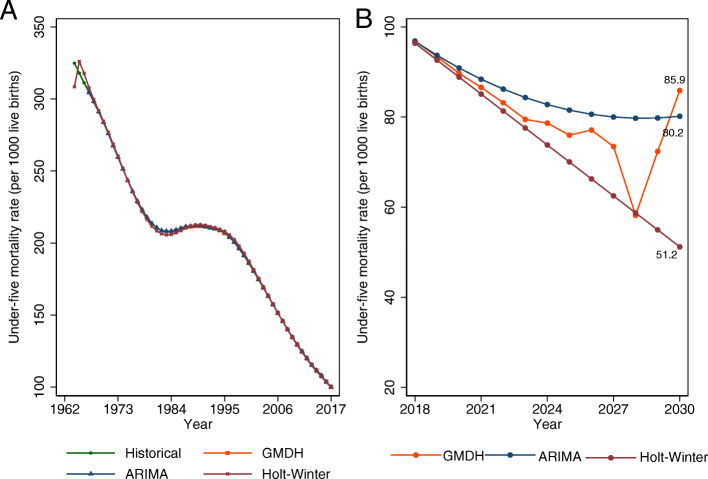
Observed (historical), predicted and forecasted under-five mortality rates by modeling techniques (**a**) in-sample prediction (1964–2017) (**b**) out-of-sample forecasting (2018–2030) *GMDH: Group method of data handling, ARIMA: autoregressive integrated moving averages, Holt-Winters: Holt-Winters exponential smoothing method. All the lines basically overlap in Plot A*

The mean annual U5MR was 203.84 (standard deviation: 58.02) deaths per 1000 live births, ranging between 324.8 deaths per 1000 live births in 1964 and 100.2 deaths per 1000 live births in 2017. From Fig. 5a, the in-sample prediction of U5MR for 1964–2017 from ARIMA, Holt-Winters and GMDH-type ANN were close to the observed historical rates. Also, similar out-of-sample rates were observed from 2018 to 2020 for the three models (Fig. 5b). However, the out-of-sample forecasts from 2021 to 2030 for each model were different (Fig. 5b). The difference is greatest for Holt-Winters model compared to GMDH-type ANN and ARIMA regression models. The forecast obtained from GMDH-type ANN model is higher than others—85.89 (95% prediction interval (PI): 85.72–86.08) deaths per 1000 live births by 2030. Holt-Winters method generated smallest mortality rate for 2030, 51.20 (95%PI: 50.66–51.73) per 1000 live births. For 2030, ARIMA model generated a rate closer to the rates for the GMDH-type ANN model—80.17 (95%PI: 79.64–80.71) deaths per 1000 live births. According to the GMDH-type model, U5MR was observed to rise from 2028 to 2030 (Fig. 5b).

The modified NSE coefficient was slightly lower for Holt-Winters methods (96.7%), compared to GMDH-type ANN (99.8%) and ARIMA (99.6%). Further comparison between GMDH-type ANN and ARIMA with RMSE, RMAE and DM test indicated that GMDH-type ANN’s performance was better (Table 1). The RMSE of GMDH-type ANN (0.09) was lower than ARIMA (0.23) and Holt-Winters (2.87). Similarly, RMAE was lowest for GMDH-type ANN (0.25), compared with ARIMA (0.41) and Holt-Winters (1.20). From the DM test, the mean absolute error (MAE) was significantly lower for GMDH-type ANN, compared with ARIMA (difference = 0.11, *p*-value = 0.0003), and Holt-Winters model (difference = 0.62, p-value< 0.001).

**Table 1:** Performance measures of time series techniques for under-five mortality rates in Nigeria

Model	GMDH-type ANN	ARIMA	Holt-Winters exponential smoothing
Best parameters	Training set = 80%, testing set = 20%	*p* = 0, d = 3, q = 2	α=0.91, β=0.51
RMSE	0.09	0.23	2.87
RMAE	0.25	0.41	1.20
Modified NSE	0.998	0.996	0.967
DM test statistic	Reference	−3.608*	−4.474*

^*^significant at p-value < 0.05; p = number of autoregressive terms, d = number of differencing, q = number of moving average terms; RMSE: Root mean squared error; RMAE: Root mean absolute error; α=coefficient for the level smoothing; β= coefficient for the trend smoothing; modified NSE: modified Nash-Sutcliffe model efficiency coefficient; DM: Diebold-Mariano test

As shown in Table 2, the coefficients of slopes and intercepts suggest there were no proportional and systematic differences between the predicted rates for the three models and the observed (historical) rates. While the slopes (proportional difference) for the three methods were similar, the intercepts (systematic difference) and standard errors were different. The lowest coefficient of intercept and SE were obtained with GMDH-type ANN (β_0_ = 0.004 ± SE: 0.06; p-value = 0.940)—implying that GMDH-type predictions were closest to the observed (historical) rates than ARIMA (β_0_ = 0.03 ± SE: 0.16; p-value = 0.865), and Holt-Winters (β_0_ = 0.89 ± SE: 2.35; p-value = 0.706).

**Table 2:** Deming regression for comparing GMDH-type ANN, ARIMA and Holt-Winters models

Reference: Observed historical U5MR	Proportional difference (slope)			Systematic difference (intercept)		
	β_1_ (SE)	95% LCL, UCL	*P*-value	β_0_(SE)	95% LCL, UCL	*P*-value
GMDH-type ANN	1.000 (0.0004)	0.999, 1.001	< 0.001	0.004 (0.058)	−0.113, 0.122	0.940
ARIMA	1.000 (0.001)	0.998, 1.002	< 0.001	0.027 (0.160)	−0.293, 0.348	0.865
Holt-Winters	1.000 (0.013)	0.969, 1.023	< 0.001	0.890 (2.349)	−3.822, 5.602	0.706

*LCL* Lower Confidence Limit, *UCL* Upper Confidence Limit, *SE* Jack-knife standard errors

## Discussion

This study compared the predictive ability of artificial intelligence technique with traditional statistical methods in view of forecasting U5MR for Nigeria from 1964 to 2030. With lowest error rates of RMAE and RMSE from this comparative analysis, we demonstrated that deep learning algorithms such as GMDH-type neural nets might be more suitable for long-term forecasting of U5MR than ARIMA regression and Holt-Winters exponential smoothing methods. Similarly, Deming regression suggests more accurate prediction of U5MR with GMDH-type ANN. Using the high efficiency coefficients (> 90%) and overlapping of the predicted rates as the criteria, all three models performed well with in-sample predictions of U5MR for Nigeria. For the period from 1964 to 2017 (in-sampling prediction) and out-of-sample forecasting from 2018 to 2020, all three models had similar results, however, for the longer out-of-sample forecasting period (2021–2030), the rates were significantly different. Also, Nigeria will not achieve child survival targets of SDG by 2030. Furthermore, GMDH-type ANN showed that U5MR will start increasing by 2028. Further analysis with age-specific mortality rates suggests that the surge in U5MR from 2028 to 2030 is due to increasing trend in neonatal mortality rates between 2028 and 2029, and child mortality rates from 2029 to 2030 (results are not shown).

In line with other studies [58, 59], our findings showed that ARIMA regression might not be suitable for long-term forecasting of U5MR, in this case for Nigeria. According to Koutsoyiannis [58, 59], ARIMA regression may not be ideal for data that exhibit long-range dependencies because of its slow decay of autocorrelation structure with lag time, making it less sensitive to tipping-points. In addition, ARIMA and Holt-Winters models assume normality of time series data, whereas under-five mortality data for Nigeria showed a non-linear trend. Also, unlike the two traditional approaches, GMDH-type ANN avoids overfitting by dropping nodes with insufficient predictive power (i.e., fully automatic structural and parametric optimization) [33]. As opposed to ARIMA and Holt-Winters methods, GMDH time series also allows for detection of recent changes in data (arising from natural behavior, policy changes and interventions), and weighs recent data more than past data during model training [29]. These detailed patterns might be easily missed by the conventional methods.

Given that more accurate results were obtained with the GMDH-type algorithm, projecting childhood mortality rates based on neural network would provide better evidence to guide prevention strategies to accelerate gains in child survival for Nigeria. A similar pattern of results was obtained from previous studies that predicted health outcomes with other artificial neural networks. Purwanto et al [60] and Zernikow et al [61] showed that multilayer perceptron ANN was superior to linear regression for predicting infant and preterm neonatal deaths, respectively.

More generally, this study indicates that, though U5MR in Nigeria continues to decline from 100.2 deaths per 1000 live births in 2017 to 85.9 deaths per 1000 live births in 2030, Nigeria might not achieve the SDG-3 target that aims to reduce the U5MR to 25 deaths per 1000 live births by 2030 [62]. More importantly, the government of Nigeria needs policy innovations to address the observed rise in U5MR by 2028. On evidence such as indicated in this paper, the government of Nigeria should use reliable estimates to improve the design and accelerate the implementation of child health programmes in order to attain the SDG-3 targets for under-five mortality by 2030.

To our knowledge, this is the first published comparative study of ARIMA, Holt-Winters and GMDH-type neural nets on childhood mortality—in Nigeria. Also, the time series used data that covered long period of time—54 years, making the models more stable. Given the high validation accuracy (93.9%) and low RMSE (0.09) of GMDH-type ANN, there is no evidence to suggest that the observed fluctuating patterns of U5MR from 2026 to 2030 is due to overfitting. Although more datapoints are needed to generate more stable models, the forecasts from this GMDH-ANN model seem adequate because of non-seasonality of the dataset [63]. As more datapoints are available in the future, it is necessary to fine-tune the GMDH-type ANN model. As often encountered with ANN modeling, a major gap is paucity of evidence for optimization of neurons for generating ANN architecture [7]. We relied on calibrations that could give maximum predictive power. Also, further assessment of GMDH-type ANN model with leave-one-out and multiple 3-fold cross-validations showed consistent findings with Pareto principle, and no sign of under/overfitting. To determine the robustness of GMDH-type ANN, further sensitivity analyses with RNN and LSTM models were performed in Jupyter notebook for Python 3 with TensorFlow interface [64]. Using Adam optimizer to train the models at different calibrations, error rates from RNN and LSTM were higher (i.e., RMSE ranged from 14 to 20), compared with GMDH-type ANN (RMSE of 0.09). In line with our observations, many studies [24, 25, 34, 35] have also demonstrated the superiority of GMDH-type ANN over both RNN and LSTM. In addition, we observed that RNN and LSTM algorithms might be less suitable because of the few data points available for this study, coupled with the problems of gradient vanishing and gradient explosion (i.e., accumulation of large error gradients leading to unstable models). To ensure generalizability, the GMDH-type ANN model was further tested on neonatal mortality, and sex-specific mortality data for Nigeria, obtained from World Bank [31]. There was no indication to suggest under(over-fitting) of data. The unexpected rise in U5MR from 2028 to 2030 warrants further investigation. It is also important to note that it is somewhat challenging to accurately estimate data preprocessing time for time series models because they are based on trial and error approach. In addition, computational time heavily relies on computer hardware efficiency such as central processing unit (CPU) and random-access memory (RAM). To generate interventions for improving child survival programmes in Nigeria, we prioritized model accuracy over time.

## Conclusions

The GMDH-type ANN predicted U5MR for Nigeria more accurately, compared to ARIMA and Holt-Winters smoothing models. Also, it does not require complicated assumptions needed for traditional time series models. The ARIMA regression and Holt-Winters methods might not be suitable for long-term forecasting of U5MR for Nigeria. Therefore, GMDH-type ANN might be more suitable for data with non-linear or unknown distribution, such as childhood mortality. GMDH-type ANN increases forecasting accuracy of childhood mortalities to inform policy actions in Nigeria and similar settings.

## Supplementary Information

**Additional file 1Table S1**. Aggregated under-five mortality rates for Nigeria, 1964–2017.

## Data Availability

Dataset for this study is attached in the Supplementary file 1.

## Abbreviations

ACF: Autocorrelation function
ANFIS: Adaptive neuro fuzzy inference system
ANN: Artificial neural network
ARIMA: Autoregressive integrated moving averages
CI: Confidence interval
CNN: Convolution neural networks
CPU: Central processing unit
DM: Diebold-Mariano test
GMDH: Group method of data handling
LMICs: Low- and middle-income countries
LSTM: Long short-term memory
MAPE: Mean absolute percentage error
MDG: Millennium Development Goal
MLP: Multilayer perceptron
MSE: Mean squared error
NSE: Nash-Sutcliffe model efficiency
PACF: Partial autocorrelation function
RAM: Radom-access memory
RMSE: Root mean squared error
RNN: Recurrent neural networks
SDG: Sustainable Development Goal
SE: Standard error
U5MR: Under-five mortality rate
UN IGME: United Nations Inter-agency Group for Child Mortality Estimation
