# Supplementary material for: Time series prediction of under-five mortality rates for Nigeria: comparative analysis of artificial neural networks, Holt-Winters exponential smoothing and autoregressive integrated moving average models
Source: BMC Med Res Methodol. 2020 Dec 3;20:292. doi: 10.1186/s12874-020-01159-9 (PMC7712624; doi:10.1186/s12874-020-01159-9)
Supplement: Supplementary file 1 — Additional file 1Table S1. Aggregated under-five mortality rates for Nigeria, 1964–2017. [file 12874_2020_1159_MOESM1_ESM.docx]

**Supporting information**

**Time series prediction of under-five mortality rates for Nigeria: comparative analysis of artificial neural networks, Holt-Winters exponential smoothing and autoregressive integrated moving average models**

*Running title: forecasting mortality with artificial intelligence*

Daniel Adedayo Adeyinka (ORCID: 0000-0003-1855-6878)^1,2^^*^, Nazeem Muhajarine (ORCID: 0000-0001-6781-5421)^1,3^

^1^Department of Community Health and Epidemiology, College of Medicine, University of Saskatchewan, Saskatoon, Canada

^2^Department of Public Health, Federal Ministry of Health, Abuja, Nigeria

^3^Saskatchewan Population Health and Evaluation Research Unit, Saskatoon, Saskatchewan, Canada

*Corresponding author: Daniel A. Adeyinka, Department of Community Health and Epidemiology, College of Medicine, University of Saskatchewan, Saskatoon, SK, S7N 5E5, Canada, Tel: +13068500086. E-mail: daa929@usask.ca

**Table S1: Aggregated under-five mortality rates for Nigeria, 1964-2017 (Source: official website of the World Bank)^(31)^**

| Year | Under-five mortality rate (per 1000 live births) |
| --- | --- |
| 1964 | 324.8 |
| 1965 | 318 |
| 1966 | 311.2 |
| 1967 | 304.7 |
| 1968 | 297.9 |
| 1969 | 291 |
| 1970 | 283.7 |
| 1971 | 275.9 |
| 1972 | 267.9 |
| 1973 | 259.7 |
| 1974 | 251.5 |
| 1975 | 243.6 |
| 1976 | 236.1 |
| 1977 | 229.2 |
| 1978 | 223.1 |
| 1979 | 217.8 |
| 1980 | 213.7 |
| 1981 | 210.6 |
| 1982 | 208.8 |
| 1983 | 208 |
| 1984 | 208.1 |
| 1985 | 208.8 |
| 1986 | 209.9 |
| 1987 | 210.8 |
| 1988 | 211.6 |
| 1989 | 212 |
| 1990 | 211.9 |
| 1991 | 211.4 |
| 1992 | 210.8 |
| 1993 | 209.9 |
| 1994 | 208.8 |
| 1995 | 206.9 |
| 1996 | 204.3 |
| 1997 | 200.7 |
| 1998 | 196.4 |
| 1999 | 191.5 |
| 2000 | 186.2 |
| 2001 | 180.6 |
| 2002 | 174.9 |
| 2003 | 169.1 |
| 2004 | 163.2 |
| 2005 | 157.3 |
| 2006 | 151.5 |
| 2007 | 145.7 |
| 2008 | 140.1 |
| 2009 | 134.7 |
| 2010 | 129.6 |
| 2011 | 124.7 |
| 2012 | 119.9 |
| 2013 | 115.6 |
| 2014 | 111.6 |
| 2015 | 107.5 |
| 2016 | 103.8 |
| 2017 | 100.2 |
